# Artificial Box C/D RNAs Affect Pre-mRNA Maturation in Human Cells

**DOI:** 10.1155/2013/656158

**Published:** 2013-03-31

**Authors:** Grigoriy A. Stepanov, Dmitry V. Semenov, Anna V. Savelyeva, Elena V. Kuligina, Olga A. Koval, Igor V. Rabinov, Vladimir A. Richter

**Affiliations:** Institute of Chemical Biology and Fundamental Medicine, Siberian Branch, Russian Academy of Sciences, Lavrentiev Avenue 8, Novosibirsk 630090, Russia

## Abstract

Box C/D small nucleolar RNAs (snoRNAs) are known to guide the 2′-O-ribose methylation of nucleotides in eukaryotic ribosomal RNAs and small nuclear RNAs. Recently snoRNAs are predicted to regulate posttranscriptional modifications of pre-mRNA. To expand understanding of the role of snoRNAs in control of gene expression, in this study we tested the ability of artificial box C/D RNAs to affect the maturation of target pre-mRNA. We found that transfection of artificial box C/D snoRNA analogues directed to *HSPA8* pre-mRNAs into human cells induced suppression of the target mRNA expression in a time- and dose-dependent manner. The artificial box C/D RNA directed to the branch point adenosine of the second intron, as well as the analogue directed to the last nucleotide of the second exon of the *HSPA8* pre-mRNA caused the most prominent influence on the level of *HSPA8* mRNAs. Neither box D nor the ability to direct 2′-O-methylation of nucleotides in target RNA was essential for the knockdown activity of artificial snoRNAs. Inasmuch as artificial box C/D RNAs decreased viability of transfected human cells, we propose that natural snoRNAs as well as their artificial analogues can influence the maturation of complementary pre-mRNA and can be effective regulators of vital cellular processes.

## 1. Introduction 

Small nucleolar RNAs (snoRNA) are commonly known to be involved in the processing of precursor ribosomal RNA (pre-rRNA) and small nuclear RNAs (snRNAs). Box C/D snoRNAs direct 2′-O-methylation of rRNA nucleotides, and box H/ACA snoRNAs guide the conversion of uridine to pseudouridine [1–4]. The snoRNA-dependent modifications are catalyzed by small nucleolar ribonucleoprotein particles (snoRNPs). Box C/D RNAs are associated with four snoRNP core proteins: NOP56, NOP58, fibrillarin, and 15.5 kDa [5–7].

**Figure 1:**
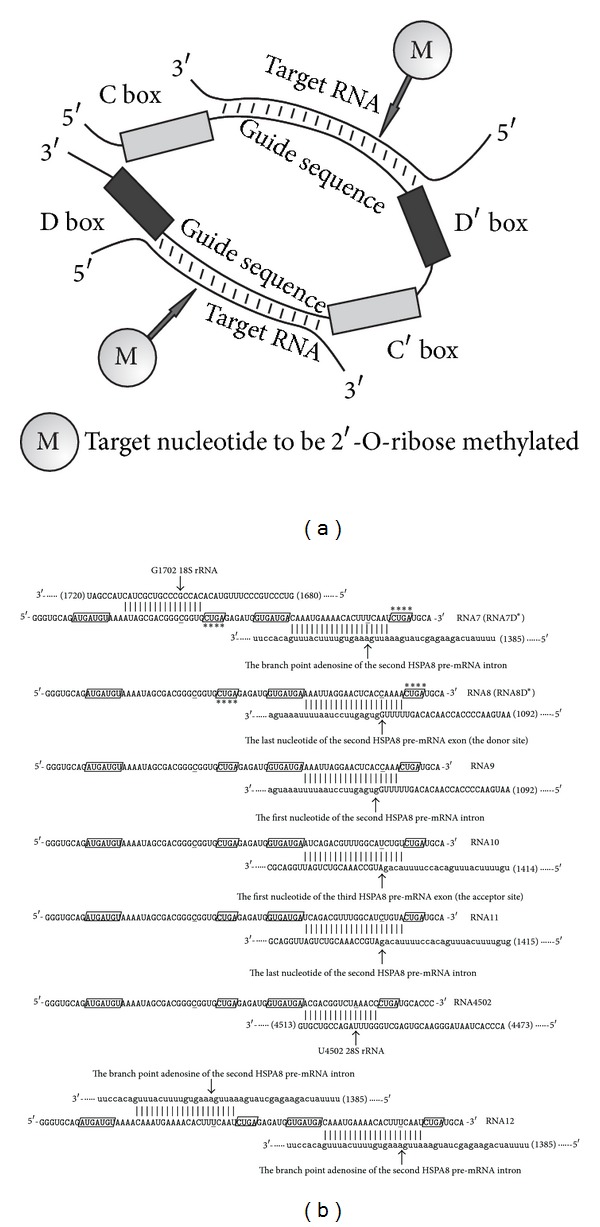
(a) Schematics of a guide box C/D snoRNA structure and the interaction between a snoRNA and target RNAs. (b) Base-pairing interactions between an artificial box C/D RNA and target RNAs. The arrows indicate potential targets of 2′-O-methylation. Uppercase letters in the target pre-mRNA sequences correspond to exons, and small letters indicate the intron sequences. Boxes C and D are indicated as **GUGAUGA** and **CUGA** enclosed in rectangles, respectively. The nucleotides to be complementary to target nucleotides are shown as **C**. RNA7D* and RNA8D* were constructed by changing the CUGA sequence in both box D and D′ to AAAA.

Box C/D RNAs contain the following functional elements: boxes C and D, which are essential for snoRNA interaction with specific proteins, and a guide sequence that determines the nucleotide to be modified (Figure 1(a)). Some box C/D snoRNAs involve a second pair of boxes C/D, named C′ and D′, within the snoRNA molecule. In that case, snoRNAs hold two potential guide sequences [2, 4].

rRNAs and snRNAs are the main natural targets of 2′-O-methylation that is mediated by box C/D RNAs. However, a number of snoRNA-like transcripts do not have guide sequences that are complementary to rRNA or snRNA, so they have unknown functions and are called “orphan snoRNAs” [8, 9]. Bioinformatics analysis revealed that some of these orphan RNAs were complementary to pre-mRNAs or mature mRNAs [10]. MBII-52 box C/D snoRNA containing a guide sequence complementary to the serotonin receptor 2C pre-mRNA was reported to take part in controlling the posttranscriptional modification of the target pre-mRNA (conversion A to I) [11]. It was also shown that HBII-52 snoRNA could regulate alternative splicing of the complementary pre-mRNA [12].

In earlier studies, the development of snoRNA expression vectors was one of the main approaches to study the influence of box C/D snoRNAs on target genes in mammalian cells. The developed DNA vectors encoded mature snoRNAs or artificial pre-mRNA fragments that were processed to snoRNA [1, 13]. Using such an approach, it was shown that introducing sequences complementary to a selected pre-mRNA into box C/D RNA structures allowed knockdown of the target mRNA [13]. In study by Ono and coworkers, such an antisense element was placed into HBII-180C box C/D RNA instead of the structural element referred to as the M box [13]. The natural M box is located within the HBII-180C snoRNA molecule and involves box C′. Replacing the M box with sequence complementary to a selected pre-mRNA allowed the snoRNA analogue to be directed to this pre-mRNA and to knockdown the mRNA target. Interestingly, the ability of the box C/D snoRNA to direct 2′-O-methylation of rRNA nucleotides was not essential for the knockdown effect [13].

Therefore, recent results indicate that box C/D RNAs that include a sequence element complementary to a pre-mRNA could control the posttranscriptional maturation of the pre-mRNA, with antisense elements located in different parts of the snoRNA molecules. In our study, we proposed and employed a novel approach to investigating the snoRNA ability to control gene expression. The approach involves direct application of artificial RNA constructions containing structural features of snoRNAs instead of DNA vectors expressing snoRNAs. Such snoRNA analogues can be addressed to different pre-mRNA or mRNA targets and delivered to the cells using transfection methods.

We selected the pre-mRNA of the *HSPA8* gene encoding the human heat shock cognate 71 kDa protein (HSC70) as a model target of snoRNA-directed action. Stress-induced proteins related to the heat shock protein 70 (HSP70) family perform numerous functions in the cell, including folding of nascent cellular proteins, regulation of intracellular transport, and directed degradation of proteins. Moreover, heat shock proteins take part in controlling repair systems, the cell cycle, cell proliferation, and differentiation [14–17]. These proteins inhibit cell death processes and cause the survival of tumorigenic cells attacked by stress factors such as cytokines, radiation, oxidative stress, and drugs, which reduces the effects of some therapeutic approaches. Therefore, controlling the expression of heat shock family genes is believed to be an option for developing complex anticancer therapy approaches [18–20]. 

Earlier, we determined that artificial U24 box C/D snoRNA analogues directed to * HSPA8 * pre-mRNA induced partial splicing impairments, namely, skipping of the exon immediately adjacent to targeted intron [21].

In this study, to further estimate the influence of box C/D RNAs analogs directed to *HSPA8* pre-mRNA on the maturation of the target pre-mRNA, we synthesized the set of artificial analogues of snoRNA. It was shown that artificial box C/D RNAs directed to *HSPA8* pre-mRNA caused not only partial splicing impairments, but also the suppression of the target mRNA expression. It was also found that the transfection of the analogues into MCF-7 human adenocarcinoma cells decreased the viability of the transfected cells.

## 2. Materials and Methods

### 2.1. Artificial Box C/D RNAs Synthesis

To design artificial box C/D RNA guide sequences, we used the *HSPA8* pre-mRNA sequence, chr11:122928200-122932901, from the GRCh37/hg19 human genome assembly and the *HSPA8* mRNA sequence from NCBI RefSeq NM_006597.4. In the text, the exons and introns of *HSPA8* pre-mRNA were numerated from the 5′-end to the 3′-end of the pre-mRNA. To identify the branch point adenosine in the second intron of *HSPA8* pre-mRNA, we used the algorithm developed by Kol et al. [22].

Synthetic analogues of box C/D RNAs were obtained via the *in vitro* transcription of PCR-amplified DNA templates with T7 RNA polymerase (Fermentas, Vilnius, Lithuania). DNA templates were synthesized using the following oligonucleotides:

 U24_Base 5′-TGCAGATGATGTAAAATAGCGACGGGCGGTGCTGAGAGATGGTGATGA-3′ (a template for DNA7, DNA8, and DNA4502);
 U24_Base_5BP 5′-TGCAGATGATGTAAAACAAATGAAAACACTTTCAATCTGAGAGATGGTGATGA-3′ (a template for DNA12);
 U24-T7 5′-ATGCAGCTAATACGACTCACTATAGGGTGCAGATGATGTAAAATAG-3′ (a universal right primer for DNA7, DNA8, and DNA4502);
 T7_5BP 5′-ATGCAGCTAATACGACTCACTATAGGGTGCAGATGATGTAAAACAAATG-3′ (a left primer for DNA12);
 24.7 5′-TGCATCAGATTGAAAGTGTTTTCATTTGTCATCACCATCTCTCAG-3′ (a right primer for DNA7 and DNA12);
 24.8 5′-TGCATCAGTTTTGGTGAGTTCCTAATTTTCATCACCATCTCTCAG-3′ (a right primer for DNA8); and
 28U4502 5′-GGGTGCATCAGGGTTTAGACCGTCGTTCATCACCATCTCTCAG-3′ (a right primer for DNA4502).

Each of the artificial RNA, namely, RNA7 (79 nt), RNA8 (79 nt), RNA9 (78 nt), RNA10 (79 nt), and RNA11 (79 nt), RNA12 (84 nt), RNA7D* (79 nt), RNA8D* (79 nt), RNA4502 (77 nt) were synthesized using corresponding DNA-template (see DNA7, DNA8, etc.).

All oligonucleotides were obtained from LMC ICBFM SB RAS, Novosibirsk. RNA transcripts were purified via ion-pair reverse phase high-performance liquid chromatography (RP-HPLC) on a Milichrome A-02 liquid chromatograph using the ProntoSIL-120-5-C18 sorbent and a 2.0 × 7.5 mm column (Econova, Novosibirsk, Russia), followed by ethanol precipitation (with 6.0 M NaOAc). RNA concentrations were determined spectrophotometrically, and RNA integrity was verified on 1.5% agarose or 5% denaturing polyacrylamide gels.

### 2.2. Transfection of MCF-7 Cells with Synthetic RNAs and Isolation of Total Cellular RNA

MCF-7 cells obtained from the Russian Cell Culture Collection of Vertebrates (Institute of Cytology, Russian Academy of Sciences, St. Petersburg, Russia) were cultured in Iscove's modified Dulbecco's medium (IMDM) (Invitrogen, Carlsbad, CA, USA) containing 10 mM L-glutamine, 40 *µ*g/mL gentamicin (Sigma-Aldrich, St Louis, MO, USA) and 10% fetal bovine serum (FBS) (Biolot, St. Petersburg, Russia).

Cells were seeded at 2 × 10^5^ cells/well in a 6-well plate and incubated 24 h in a humidified incubator at 37°C with 5% CO_2_. Synthetic analogues of box C/D RNAs were preincubated with the Lipofectamine reagent (Invitrogen) according to the manufacturer's protocol and were added to the culture medium in the concentration indicated in the figure legends. After incubating the cells with the analogs, total RNA was isolated using the Trizol reagent (Invitrogen) according to the manufacturer's protocol.

### 2.3. Accumulation of Radiolabeled Artificial Box C/D RNAs in Human Cells

Synthetic RNAs were 5′-[^32^P]-labeled with T4 polynucleotide kinase (Biosan, Novosibirsk, Russia). RNA forms were separated on a 10% denaturing polyacrylamide gel, and individual RNA forms were eluted from gel bands into 0.3 M NaOAc. Transfection of radiolabeled RNAs into human cells was performed as described above. The transfected cells were cultured for time intervals ranging from 3 h to 72 h. Total RNA was isolated with Trizol and analyzed in a 10% denaturing polyacrylamide gel followed by autoradiography of radiolabeled RNA fragments.

### 2.4. Flow Cytometry Analysis

MCF-7 cells (1 × 10^6^) in 6-well plates were transfected with 160 nM FAM-labeled (FAM—carboxyfluorescein) artificial RNA. Control cells were incubated with Lipofectamine only. After incubation for 24  hours, the cells were detached with 0.025% trypsin and washed twice with IMDM containing 10% FBS. The transfected cells were scanned using a FACSCanto II flow cytometer (BD Biosciences, Franklin Lakes, NJ, USA) and the data were analyzed by FACSDiva Software (BD Biosciences).

### 2.5. RT-PCR

The reverse transcription and cDNA amplification (RT-PCR) were performed in the one-tube reaction mixture “Real Best Master Mix RT” (Vektor-Best, Novosibirsk, Russia) with the addition of Sybr Green (Biodye, Moscow, Russia) in the case of real-time RT-PCR. The following gene-specific primers were used: *HSPA8*: hsp-2.1 5′-ACTGAACGGTTGATCGGTGA-3′ and hsp-8.2 5′-AGATGAGCACGTTTCTTTCT-3′; *HPRT*: HPRT_1 5′-CATCAAAGCACTGAATAGAAAT-3′ and HPRT_2 5′-TATCTTCCACAATCAAGACATT-3′; *GAPDH*: GAPDH-1 5′-GAAGATGGTGATGGGATTTC-3′ and GAPDH-2 5′-GAAGGTGAAGGTCGGAGT-3′; and 28S rRNA: 28-2.1 5′-TAGACCGTCGTGAGACAGGT-3′ and 28-2.2 5′-ATTGGCTCCTCAGCCAAGCA-3′. To compare PCR product yields, we carried out real-time RT-PCR (on Bio-Rad iQ5 Cycler, Hercules, CA, USA). The data were analyzed using iQ5 system software (Bio-Rad). Mean values (±SD) from 3 independent experiments were represented. The PCR products were analyzed in a 4% denaturing polyacrylamide gel or 1.5% agarose gel.

### 2.6. MTT Assay

To estimate the influence of the artificial box C/D RNA on MCF-7 human adenocarcinoma cell viability and proliferation we used MTT assay (MTT (3-(4,5-dimethyl-2-thiazolyl)-2,5-diphenyl-2Htetrazolium bromide), Sigma-Aldrich). Aliquots (150 mL) containing 2.5 × 10^3^ cells were plated onto 96-well plates and incubated at 37°C with 5% CO_2_. After 24 h, the cells were transfected with 40–160 nM artificial RNAs. After incubation for 72 h, MTT was added to the cells at a final concentration of 0.5 mg/mL and the plates were incubated at 37°C for 3 h. The medium was removed, followed by the addition of 0.15 mL of DMSO to each well. The plates were read at 570 and 620 nm using an Apollo LB912 plate reader (Berthold Technologies, Oak Ridge, TN, USA). Cell viability was determined as the absorbance at 570 nm with reference to 620 nm and expressed as a percentage of control (control cells were incubated with Lipofectamine only) ±SD for triplicate independent experiments.

## 3. Results and Discussion

### 3.1. Structure of Artificial Box C/D RNAs

To investigate the influence of box C/D RNA on pre-mRNA maturation, we constructed and synthesized U24 snoRNA analogues containing guide sequences complementary to *HSPA8 *pre-mRNA. Human U24 is a 76 nt long conserved RNA which contains two pairs of C/D boxes (C, D, C′, D′). It is predicted to guide the 2′O-ribose methylation of both 28S rRNA C2338 and C2352 [23, 24].

Analogues were constructed to direct 2′-O-methylation of G1702 in 18S rRNA and of the *HSPA8* pre-mRNA nucleotides that were critical to splicing the second intron of the pre-mRNA-target (Figure 1(b)). To obtain the artificial box C/D RNAs, we constructed a series of DNA templates and primers containing the T7 promoter. The templates were amplified, and the PCR-products were used as the templates for *in vitro* transcription with T7 RNA polymerase.

The artificial RNAs had two pairs of C/D boxes (C, D, C′, D′) and two guide sequences. The first guide sequence (from the 5′-terminus) was directed to G1702 in 18S rRNA, which was one of the key nucleotides of the ribosome-decoding center [25]. Earlier, we studied the changes in the 18S rRNA structure after transfection of artificial box C/D RNAs into human cells. The analysis revealed that artificial RNAs induced the addition of reverse transcription termination sites on rRNA, whereas the snoRNA analogues did not cause 2′-O-methylation of the target nucleotide [21]. The second guide sequence of the artificial RNAs was targeted to the *HSPA8* pre-mRNA nucleotides that were critical to splicing the second intron, namely, the branch point adenosine in the second *HSPA8* pre-mRNA intron (RNA7), the last nucleotide of the second exon (RNA8), the first and the last nucleotides of the second intron (RNA9 and RNA11, respectively), and the first nucleotide of the third exon of the target pre-mRNA (RNA10, Figure 1(b)). Artificial box C/D RNA designated as RNA12 contained two guide sequences directed to the branch point adenosine in the second *HSPA8* pre-mRNA intron (Figure 1(b)). Analogues RNA7D* and RNA8D*, where both the box D and D′ CUGA sequences were replaced with AAAA (Figure 1(b)), were targeted to the branch point adenosine in the second intron and the last nucleotide of the second exon of *HSPA8* pre-mRNA, respectively.

We have also obtained box C/D RNA analogue directed to two rRNA nucleotides, namely, G1702 in 18S rRNA and U4502 in 28S rRNA (RNA4502, Figure 1(b)). In other studies, we investigated the influence of such analogues on human cells [21]. Here we used RNA4502 as control RNA that was not complementary to the *HSPA8* pre-mRNA.

### 3.2. Accumulation of Artificial Box C/D RNAs in Transfected Human Cells

To investigate the efficiency of artificial box C/D RNAs transfection into human cells, we synthesized FAM-labeled RNA analogues and studied the internalization and compartmentalization of the RNAs in MCF-7 cells via fluorescence microscopy. It was found that the fluorescent RNAs were detected in the cytoplasm as well as in nuclei. These results were confirmed with RT-PCR of nuclear and cytoplasmic total RNA from MCF-7 cells transfected with the artificial box C/D RNA [21].

**Figure 2:**
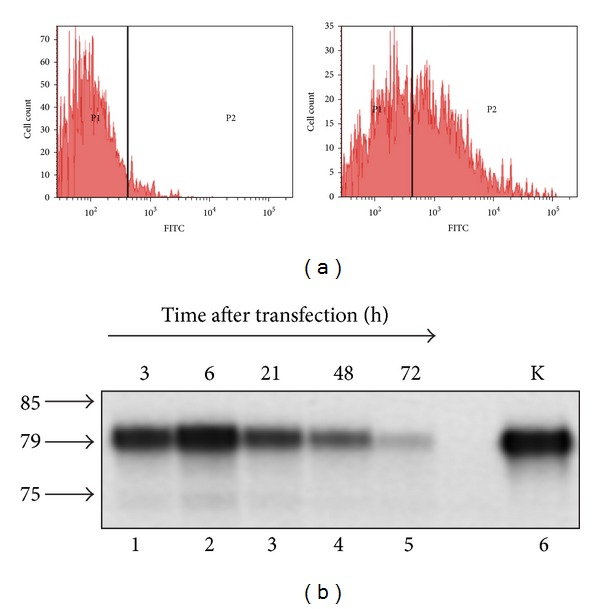
(a) Accumulation of FAM-labeled artificial box C/D RNAs in human cells. MCF-7 cells transfected with FAM-labeled RNA8 analogue using Lipofectamine were analyzed with flow cytometry. “P2” population represents MCF-7 cells positively stained with FAM-labeled RNA, with “P1” population representing nonstained cells. (b) Presence of radiolabeled artificial box C/D RNA in total RNA from transfected MCF-7 cells. Radiolabeled RNA8 was transfected into cells using Lipofectamine, and the cells were cultured for time intervals from 3 h to 72 h (lanes 1–5). Total cellular RNA was isolated and analyzed in a 10% denaturing polyacrylamide gel followed by autoradiography of the radiolabeled RNA fragments. The RNA loading was normalized by spectrometry, and 3 *µ*g of total cellular RNA was used per lane. Lane 6 intact radiolabeled artificial box C/D RNA. The arrows indicate marker mobility (in parallel sequencing reaction product were loaded on the same gel).

Here, to estimate the efficiency of transfection of box C/D RNA into human cells we treated MCF-7 cells with artificial FAM-labeled RNA in complex with Lipofectamine and analyzed cell populations using flow cytometry. The analysis of cells transfected with FAM-RNA8 revealed that 50% of the treated cells reliably contained the fluorescent snoRNA analogue (Figure 2(a)). The similar results were obtained for the complete set of box C/D RNA analogues used.

Analysis of radiolabeled box C/D RNA analogue accumulation and integrity in human cells showed that full-length RNA (79 nt long) molecules were detected in the transfected cells at least 72 h after RNA transfection (in complex with Lipofectamine, Figure 2(b)). Thus, we concluded that artificial box C/D RNAs effectively accumulated and persisted in human cells at least 72 h under the transfection conditions used.

### 3.3. Artificial Box C/D RNAs Affect the Maturation of Complementary Pre-mRNA

**Figure 3:**
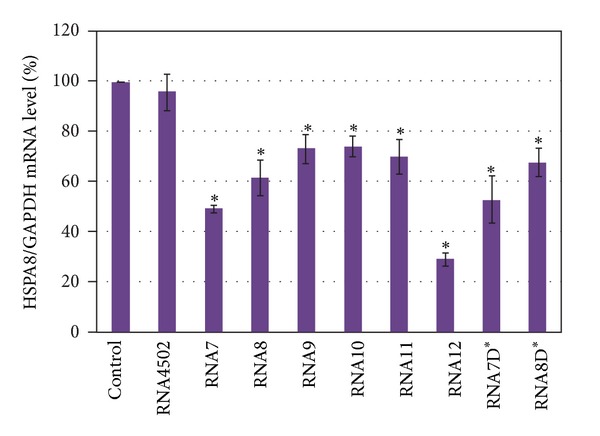
Level of *HSPA8* mRNA 24 h after transfection of artificial box C/D RNAs into MCF-7 cells (160 nM RNA in culture medium). MCF-7 human cells were transfected with RNA7, RNA8, RNA9, RNA10, RNA11, RNA12, RNA7D*, RNA8D* directed to *HSPA8 *pre-mRNA and RNA4502 directed to ribosomal RNAs. Control cells were incubated with Lipofectamine only. The level of mRNA was determined with quantitative RT-PCR and was represented as relative values normalized to the level of GAPDH mRNA. The error bars represent standard deviations. The asterisks (*) indicate significant difference from control (*P* < 0.05).

We transfected artificial box C/D RNAs (160 nM RNA complexed with Lipofectamine in culture medium) into MCF-7 human mammary adenocarcinoma cells, isolated total RNA 21 h after transfection, and performed RT-PCR with primers specific to *HSPA8* mRNA. We found that transfection of the artificial analogues into MCF-7 human cells suppressed the *HSPA8* mRNA level. As shown in Figure 3, the artificial U24 box C/D RNA directed to the branch point adenosine of the second intron of the *HSPA8* pre-mRNA (RNA7) and to the last nucleotide of the second exon of the *HSPA8* pre-mRNA (RNA8) caused the decrease in target mRNAs level by about 50% and 40%, respectively. In the same conditions, box C/D RNA analogues directed to other nucleotides in the *HSPA8* pre-mRNA (the first and the last nucleotides of the second intron (RNA9 and RNA11, respectively), and the first nucleotide of the third exon of the target pre-mRNA (RNA10, Figure 1(b))) had smaller effects, which were less than 30%.

Interestingly, the insertion of additional complementary elements to the artificial box C/D RNA structure strengthened the suppression effect. Box C/D RNA analogues having two identical guide sequences directed to the branch point adenosine of the second intron of the *HSPA8 *pre-mRNA (RNA12, Figure 1(b)) intensified the downregulation effect on the target mRNA level (1.7–2.2 times) compared to the effect from RNA7 (Figure 3).

**Figure 4:**
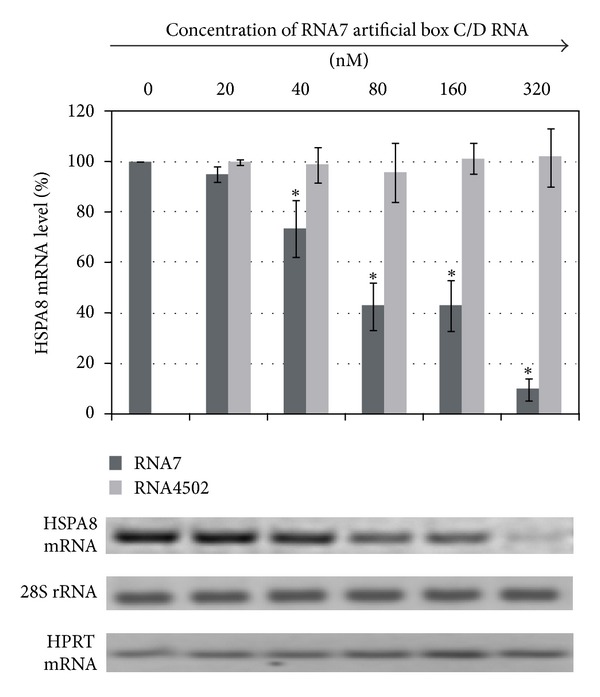
Level of *HSPA8* mRNA after RNA7 transfection into MCF-7 cells. The indicated concentrations of RNA7 were transfected into cells using Lipofectamine, and the cells were cultured for 21 h. Control cells (0 nM RNA7) were incubated with Lipofectamine only. The error bars represent standard deviations. The asterisks (*) indicate significant difference from control (*P* < 0.05).

**Figure 5:**
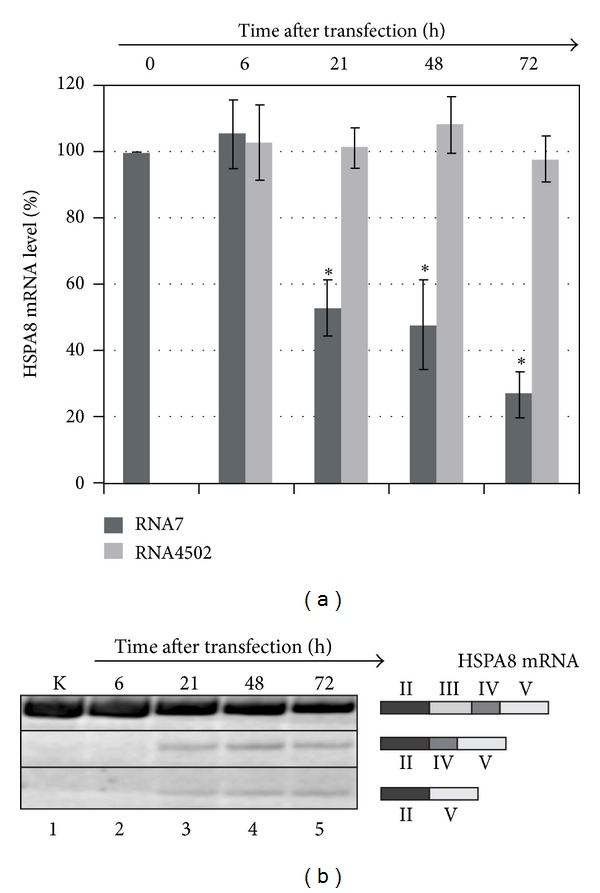
(a) Time course of the *HSPA8* mRNA RNA level in MCF-7 cells treated with artificial box C/D RNAs. RNA7 (160 nM) was transfected into cells using Lipofectamine, and then the cells were cultured for time intervals from 6 h to 72 h. Control cells (indicated as “0 h”) were incubated with Lipofectamine only. The error bars represent standard deviations. The asterisks (*) indicate significant difference from control (*P* < 0.05). (b) Splicing variants of *HSPA8* pre-mRNA after transfection of artificial box C/D RNA targeted to the branch point adenosine into MCF-7 cells. RNA7 (160 nM) was transfected into cells using Lipofectamine. Cells were cultured for the time indicated. Control cells were incubated with Lipofectamine only. The schematic structures of splicing forms are represented on the left, with II, III, IV, and V corresponding to the numbers of *HSPA8* mRNA exons.

We next examined the time- and concentration-dependence of *HSPA8* mRNA suppression. The higher the concentration of box C/D RNA targeted to the branch point adenosine of the second intron of the *HSPA8 *pre-mRNA during transfection, the lower the *HSPA8* mRNA level (up to 10%) in MCF-7 cells (Figure 4). Increasing the transfection time from 6 h to 72 h reduced the *HSPA8* mRNA amount to 30% of its original level (a 3-fold decrease) (Figure 5(a)).

The decreased yield of the major product of *HSPA8*-specific RT-PCR was accompanied by increased yield of minor products corresponding to aberrant (shorter) products of *HSPA8* pre-mRNA splicing. The primary structures of RT-PCR products were determined with Sanger sequencing and are schematically represented in Figure 5(b). The detected products were determined to be *HSPA8* mRNA splicing variants that resulted from skipping the second or simultaneously skipping both the third and fourth exons of the major mRNA isoform. The skipping of exons resulted in a shift in the mRNA reading frame and the appearance of premature stop-codons. It can be supposed that the aberrant splice variants undergo the rapid degradation through the nonsense-mediated mRNA decay pathway [26, 27].

The directional influence on the pre-mRNA splicing is one of the promising approaches for gene expression modulation. Such an approach has some advantages; for instance, it provides control of the gene expression in stages of mRNA maturation prior to mRNA appearance in the cytoplasm [28–30]. Thus, our approach implying the use of artificial snoRNA analogues affecting the splicing and overall mRNA isoform levels may be a prospect for the development of novel therapeutic approaches.

### 3.4. The Effect of Box C/D RNA Analogues on the Viability of Human Adenocarcinoma MCF-7 Cells

**Figure 6:**
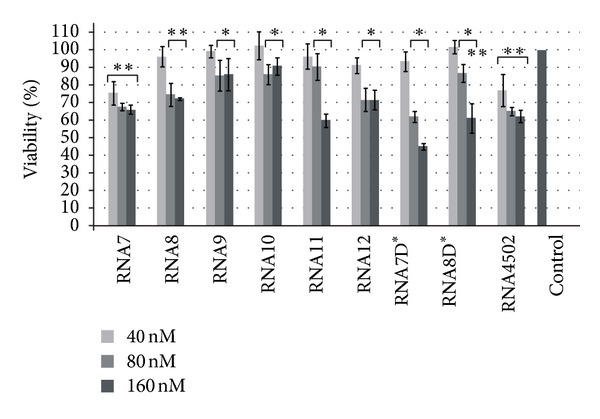
The effect of box C/D RNA analogues on the viability of human adenocarcinoma MCF-7 cells. Cells were transfected with 40 nM, 80 nM, and 160 nM artificial RNA (in complex with Lipofectamine) for 72 h and viability was analyzed by MTT assay. Control cells were incubated with Lipofectamine only. Data are presented as the mean of at least three separate experiments. The error bars represent standard deviations. The asterisks (* or **) indicate significant difference from control (*P < 0.05 and **P < 0.01).

We also estimated the influence of the artificial box C/D RNA on MCF-7 cell viability and proliferation using the MTT assay. We found that box C/D RNA analogues directed on nucleotides in *HSPA8* pre-mRNA decreased MCF-7 viability (Figure 6). Some interrelationship between an ability of a particular snoRNA to decrease *HSPA8* mRNA level and its effect on cell viability was found. For instance, the artificial box C/D RNAs directed to the branch point adenosine of the second intron of the *HSPA8* pre-mRNA (RNA7, RNA12, and RNA7D*) caused the most prominent influence on MCF-7 viability and reduced MTT-index by more than 28% at a final concentration of artificial RNAs 80 nM (Figure 6). As shown above the same set of artificial box C/D RNA induced the most conspicuous influence on the level of *HSPA8* mRNAs (Figure 3).

When RNA concentration was increased up to 160 nM correlation between *HSPA8* mRNA suppression effect and MCF-7 viability reduction was not observed (Figure 6). Moreover, as it is seen from Figures 2 and 6, RNA4502 did not induce appreciable suppression of the *HSPA8 *mRNA expression but nevertheless reduced MCF-7 viability (by 22%, 35%, and 37% at 40 nM, 80 nM, and 160 nM, resp., Figure 6). These facts suggested that in addition to the decrease in the *HSPA8 *mRNA level there was some accessory process affecting the viability of transfected human cells. It is most likely that accompanying process induced in response to artificial box C/D RNA was activation of the innate immune system in transfected cells. The combination of the gene-specific knockdown and the activation of the interferon response system were reported earlier for a small interfering RNA-dependent regulation of gene expression [31–33].

### 3.5. Complementary Interaction with the Target Pre-mRNA is the Key Factor for the Knockdown Ability of Box C/D RNA Analogues

The questions of whether box C/D snoRNAs direct 2′-O-methylation during their interaction with pre-mRNAs and whether modification of the target nucleotide is essential for the ability of the snoRNA to regulate gene expression remain open. Classical methods to detect 2′-O-methylated RNA nucleotides do not reveal modified nucleotides in minor RNA forms such as pre-mRNA. At the same time, box D is known to be an essential element for snoRNA-guided 2′-O-methylation.

To study the participation of box D of artificial RNAs in pre-mRNA knockdown activity, we constructed U24 RNA analogues RNA7D* and RNA8D*, where both the box D and D′ CUGA sequences were replaced with AAAA (Figure 1(b)). RNA7D* and RNA8D* were targeted to the branch point adenosine in the second intron and the last nucleotide of the second exon of *HSPA8* pre-mRNA, respectively.

We found that transfection of RNA7D* or RNA8D* into human cells decreased the *HSPA8* mRNA level to 53 ± 9.3% and 68 ± 5.6%, respectively (Figure 3). The decrease in targeted mRNA expression induced by RNAs with a mutated D-box RNAs was practically the same as the decrease in expression caused by the nonmutated counterparts. This result indicated that the mutation of box D in artificial RNA did not abolish the HSPA8 mRNA suppression effect. Our results showed that neither the box D structural element (CUGA) nor the ability to direct 2′-O-methylation of nucleotides in target RNA were essential for the knockdown activity of artificial snoRNAs. Thus, complementary interaction with the target pre-mRNA was supposed to be the main pathway of artificial RNA influence on the target pre-mRNA maturation.

There are numerous RNA transcripts that are complementary to different pre-mRNAs in eukaryotic cells, but their functions are not well understood. Antisense transcripts could take part in a variety of vital processes including chromatin remodeling, regulation of transcription, posttranscriptional modifications, and intracellular transport [34–37].

Our results suggest that posttranscriptional pre-mRNA maturation could be affected by artificial analogues of snoRNAs with antisense sequences that are complementary to pre-mRNAs. Furthermore, our results suggest that the observed reduction of mRNA level occurs independently of the D/D′ boxes of the analogues and thus independently of their potential ability to guide 2′-O-methylation of the target pre-mRNA.

## 4. Conclusion

The study provides a novel tool to offer insights into the question of whether snoRNAs that are complementary to pre-mRNAs could affect the processes of target pre-mRNA maturation. Our results showed that transfection of human cells with artificial snoRNAs containing guide sequences directed to pre-mRNA could impair the splicing and affect overall mRNA isoform levels. Transfection with artificial snoRNAs decreased human cell viability, suggesting that snoRNA analogues are able to activate regulatory processes affecting vital cellular functions.
